# Integrating Spatial Transcriptomics and Single-nucleus RNA Sequencing Reveals the Potential Therapeutic Strategies for Uterine Leiomyoma

**DOI:** 10.7150/ijbs.83510

**Published:** 2023-05-08

**Authors:** Jianzhang Wang, Ping Xu, Gen Zou, Xuan Che, Xiaohong Jiang, Yuanmeng Liu, Xinqi Mao, Xinmei Zhang

**Affiliations:** 1 Department of Gynecology, Women's Hospital, School of Medicine, Zhejiang University, Hangzhou, Zhejiang, P.R. China, 310006.; 2 Zhejiang Provincial Key Laboratory of Precision Diagnosis and Therapy for Major Gynecological Diseases, Women's Hospital, Zhejiang University School of Medicine, Hangzhou, Zhejiang, P.R. China, 310006.; 3 Department of Gynecology, Jiaxing University Affiliated Women and Children Hospital, Jiaxing, Zhejiang, P.R. China, 314000.

**Keywords:** Uterine leiomyoma, Pseudocapsule, Spatial transcriptomics, Single-cell RNA sequencing, Bleeding control, prostaglandin E2

## Abstract

Uterine leiomyoma is the most common gynecological tumor in reproductive women. Tumor-host interface is a complex ecosystem with intimate cell-cell communications and a critical scenario for tumor pathogenesis and progression. The pseudocapsule is the main tumor-host interface of uterine leiomyoma, but its cellular spatial disposition and gene expression are poorly explored. This study mapped the cellular architecture and corresponding gene profiles of the leiomyoma and its surrounding pseudocapsule by integrating spatial transcriptomics and single-nucleus RNA-sequencing at the first time. Here, we reported that estrogen receptor alpha and progesterone receptor mediated the occurrence and development of uterine leiomyoma and that estrogen receptor beta involved in the angiogenesis, which explained the effectiveness of hormonotherapy. Therapeutic targets including ERK1/ERK2 pathway and IGF1-IGF1R were found and might be applied for non-hormonal therapy of uterine leiomyoma. Furthermore, the injection of prostaglandin E2 was initially presented for bleeding control during myomectomy, injection site should be located at the junction between pseudocapsule and leiomyoma, and surrounding pseudocapsule should not be eliminated. Collectively, a single-cell and spatially resolved atlas of human uterine leiomyoma and its surrounding pseudocapsule was established. The results revealed potentially feasible strategies for hormonotherapy, non-hormonal targeted therapy and bleeding control during myomectomy.

## Introduction

Uterine leiomyoma (myoma or fibroid) is the most prevalent benign tumor of genital organs in the women of reproductive age, with increasing incidence rate 1, 2. It can be detected in 51% of premenopausal women by ultrasonographic screening 3 and in 77% of uteri after hysterectomy 4. More than 30% of patients suffer from heavy menstrual bleeding, infertility, pelvic pain and pregnancy complications. This disease leads to 40-60% of all the performed hysterectomies worldwide, causing greatly harm to the patients 5. Until now, there is no effective drug for the treatment of uterine leiomyoma, and myomectomy is the most common therapy method for the patients with symptoms 6. One of reasons is lack of elucidation of the precise cellular and molecular changes in the pathogenesis and development of uterine leiomyoma. To gain more knowledge regarding to its underling mechanism and spatial disposition will contribute to the treatment of uterine leiomyoma.

Currently, single-cell RNA sequencing (scRNA-seq) serves as a novel technology analyzing the transcriptomes of a complex tissue at single-cell resolution and has brought tremendous benefits to the medical research. scRNA-seq can detect the specific cell types, cell-cell interactions and epigenetic factors and overcome many disadvantages of bulk RNA sequencing 7. As for the application of scRNA-seq in uterine leiomyoma, there is only one study by a MEDLINE and PubMed search. Goad et al. depicted a cellular atlas for MED12-variant positive leiomyoma and myometrium and revealed the unknown heterogeneity in the smooth muscle cells, fibroblast and endothelial cell populations of uterine leiomyoma and myometrium 8. However, scRNA-seq requires the single cell suspension and thereby the dissociation of targeted tissue causes a total loss of cellular spatial disposition, which is critical to make clear the pathological changes of diseases 9. Therefore, a comprehensive interpretation of how and where targeted cells interact with surrounding microenvironment is lacking, and new technologies deserve further investigation.

Spatial transcriptomics (ST), a next generation tool for tissue exploration, has emerged to address the limitations of scRNA-seq by preserving tissue architecture. Since ST can draw specific cellular maps of tissue architecture and their location in relation to surrounding cells, it has been widely used in the study of various diseases especially in cancers and helped us better understand their underlying mechanisms of pathogenesis and progression 10. Here, we first applied the ST technology to the research of uterine leiomyoma. The cellular resolution of ST is usually lower than that of scRNA-seq, so ST and scRNA-seq can be integrated to maximize respective benefits and avoid separate drawbacks. Given that single-nucleus RNA sequencing (snRNA-seq) has its own advantages especially for muscle cells and with ST integration 11-13, the snRNA-seq and ST was combined to better investigate uterine leiomyoma in this study.

Tumor-host interface is a microenvironment where tumor cells emerge from and a microecology in which cell-cell communications take place 14, 15. Therefore, the interface is always a critical scenario for the progression of diseases. The pseudocapsule is the main interface of uterine leiomyoma and is a relatively loose structure with rich vascular network that separates the leiomyoma from normal myometrium 16. Our knowledge about its role on the pathogenesis, angiogenesis and progression of uterine leiomyoma is limited. ST is a sharp sword to study the junction between targeted lesion and surrounding tissue so we would pay close attention to the leiomyoma and its surrounding pseudocapsule. Here, we mapped the cellular architecture and exhibited the gene expression profiles of the leiomyoma, the surrounding pseudocapsule and normal myometrium by integrating ST and snRNA-seq.

## Materials and methods

### Sample preparation

This project obtained approval from the Ethics Committee of Women's Hospital, School of Medicine, Zhejiang University (IRB-20220297-R). Written informed consent was acquired from included patients for the collection of specimens and information use. Four samples including one for ST and three for snRNA-seq were obtained from a 38-year-old woman with uterine leiomyoma during the hysterectomy. The targeted tissue included a part of uterine leiomyoma, its surrounding pseudocapsule and normal myometrium in the distance. And the tissue was then cut into two parts and one part was used for ST (named as P1-ST). In another counterpart to ST, leiomyoma (labeled as P1L-SC), surrounding pseudocapsule (labeled as P1P-SC) and normal myometrium (labeled as P1N-SC) were separated and subjected to snRNA-seq respectively. Three samples including one for ST and two for snRNA-seq were obtained from another 44-year-old patient with uterine leiomyoma during the hysterectomy. The targeted tissue included a part of uterine leiomyoma and its surrounding pseudocapsule. And the tissue was then cut into two parts and one part was used for ST (named as P2-ST). In another counterpart to ST, leiomyoma (labeled as P2L-SC) and surrounding pseudocapsule (labeled as P2P-SC) were separated and subjected to snRNA-seq respectively.

### Frozen embedded tissue and slide preparation for 10X Visium Spatial transcriptomics

The tissues were cut into small pieces (6.5 mm^3^), snap-frozen in isopentane, pre-chilled with liquid nitrogen, embedded in optimum cutting temperature compound, cut at 10 μm thickness in a pre-cooled cryostat, systematically placed on chilled Visium Tissue Optimization slides and Visium Spatial Gene Expression Slides (10x Genomics, Pleasanton, CA, USA), and stored at -80°C until use. The spatial transcriptomics slides were printed with four identical 6.5×6.5 mm capture areas, and each area contained 5,000 spots with barcoded primers. The spots were arranged in a centeredregular hexagonal grid with a diameter of 55 μm so that each spot has six surrounding spots with a center-to-center distance of 110 μm.

### Fixation, staining, imaging, spatial library preparation and sequencing for 10X Visium Spatial transcriptomics

Sectioned slides were incubated at 37°C for 1 minute, and then fixed in 3.7-3.8% formaldehyde for 30 minutes. After washing, sections were incubated in Mayer's hematoxylin for 4 minutes, and Eosin for 30 seconds. Following, the slides were mounted with 85% glycerol (Merck Millipore, Burlington, MA) for bright-field imaging at 20× magnification using Metafer Slide Scanning platform. For pre-permeabilization, sections were incubated with 0.5 U/ml collagenase and 0.2 mg/ml bovine serum albumin (BSA) in hanks' balanced salt solution (HBSS) buffer at 37°C for 20 minutes. Permeabilization was then incubated in 0.1% pepsin (Sigma-Aldrich, Missouri, USA) dissolved in 0.1M HCl at 37°C for 7 minutes. Reverse transcription was performed as previously reported 17. Slides were then incubated in a tissue removal mix of Proteinase K (QIAGEN, Stockach, German) at 56°C with interval shaking for 1.5 hours. The spatially barcoded cDNA was enzymatically released as reported 17. Second-strand cDNA synthesis was performed on-slide for 15 minutes at 65°C in a thermocycler after supernatants with the released cDNA were carefully collected. After the cDNA amplification and cleanup of the above samples, the libraries were diluted to 4 nM and sequenced on the Illumina NovaSeq 6000 using paired-end sequencing (Biomarker Technologies Corporation, Beijing, China).

### Data analysis for 10X Visium Spatial transcriptomics

For the upstream analysis, sequencing data was processed using the Space Ranger pipeline v.1.2.2 (10x Genomics). The mapping was performed based on GRCh38_release 95 human genome as the reference. The count matrixes and spatial data were obtained for the downstream analysis. Data were processed using R version 4.0.3 and Seurat package 4.0.2 and normalized by SCTransform. Principal component analysis (PCA) and t-distributed stochastic neighbor embedding (t-SNE) dimensionality reduction were conducted using default parameters. Initial clustering was done using the FindClusters function with the resolution parameter = 0.6 for three samples (P1L-SC, P1P-SC and P1N-SC) and 0.8 for two samples (P2L-SC and P2P-SC). Marker genes were screened using Findmarkers, and visualization was done through R.

### Tissue dissociation and nucleus isolation for 10x Genomics snRNA-seq

Nucleus isolation was conducted as previously described 18. Briefly, each tissue was cut into small pieces and homogenized with a glass Dounce tissue grinder. The tissue was homogenized for 25 times with pestle A and B in 2 ml of ice-cold nuclei EZ lysis buffer (Sigma-Aldrich). After five-minute incubation on ice, nuclei were centrifuged at 500g for 5 minutes. After centrifugation, the above nucleus pellet was further washed with 5 ml nuclei suspension buffer. Isolated nuclei were resuspended, filtered through a 35 μm cell strainer and counted. A final concentration of 1,000 nuclei per μl was obtained for further experiment.

### snRNA-seq library preparation and sequencing

The nuclei suspension was loaded onto the Chromium Next GEM Chip G and single-cell gel beads were generated in the Gel Bead-in-emulsion (GEMs) using Chromium Controller according to the manufacturer's recommendation. Captured nuclei in single-cell gel beads were lysed and the released RNA was then barcoded by reverse transcription in individual GEMs. Barcoded and full-length cDNA was synthesised and libraries were constructed. The quality of libraries was controlled by Qubit 4.0 and the Agilent 2100. Sequencing was conducted on the Illumina NovaSeq 6000 with a depth of at least 50,000 reads per nucleus and 150 bp (PE150) paired-end reads (Biomarker Technologies Corporation, Beijing, China).

### Data analysis for 10x Genomics snRNA-seq

The sequencing data was processed using the 10x Cell Ranger v7.0 and the reference genome was the human genome GRCh38. Overall, 26,371 nuclei passed the quality control. R version 4.0.3 and Seurat package 4.0.2 were applied for the data process. For quality control, nuclei with gene number from 200 to 4000 and the ratio of mitochondria lower than 5 were maintained. The steps including normalization, PCA and t-SNE analysis were performed for the dimensionality reduction and cell clustering. After the screening of marker genes by the Seurat function FindAllMarkers, the combination SingleR (v1.4.1) and cell Marker database was applied for cell type annotations. The differentially expressed genes (DEGs) were identified using the FindMarkers function in Seurat. Enrichr 19 and MSigDB Hallmark gene sets 20 were used to perform gene set enrichment analysis against the Gene Ontology (GO) Biological Process 2018 version gene set collection. Monocle 2 was applied for placing cells onto pseudotime trajectories as previously described 21.

## Results

### The morphology and spatial transcriptomics of human uterine leiomyoma and its surrounding pseudocapsule

**Figure 1:**
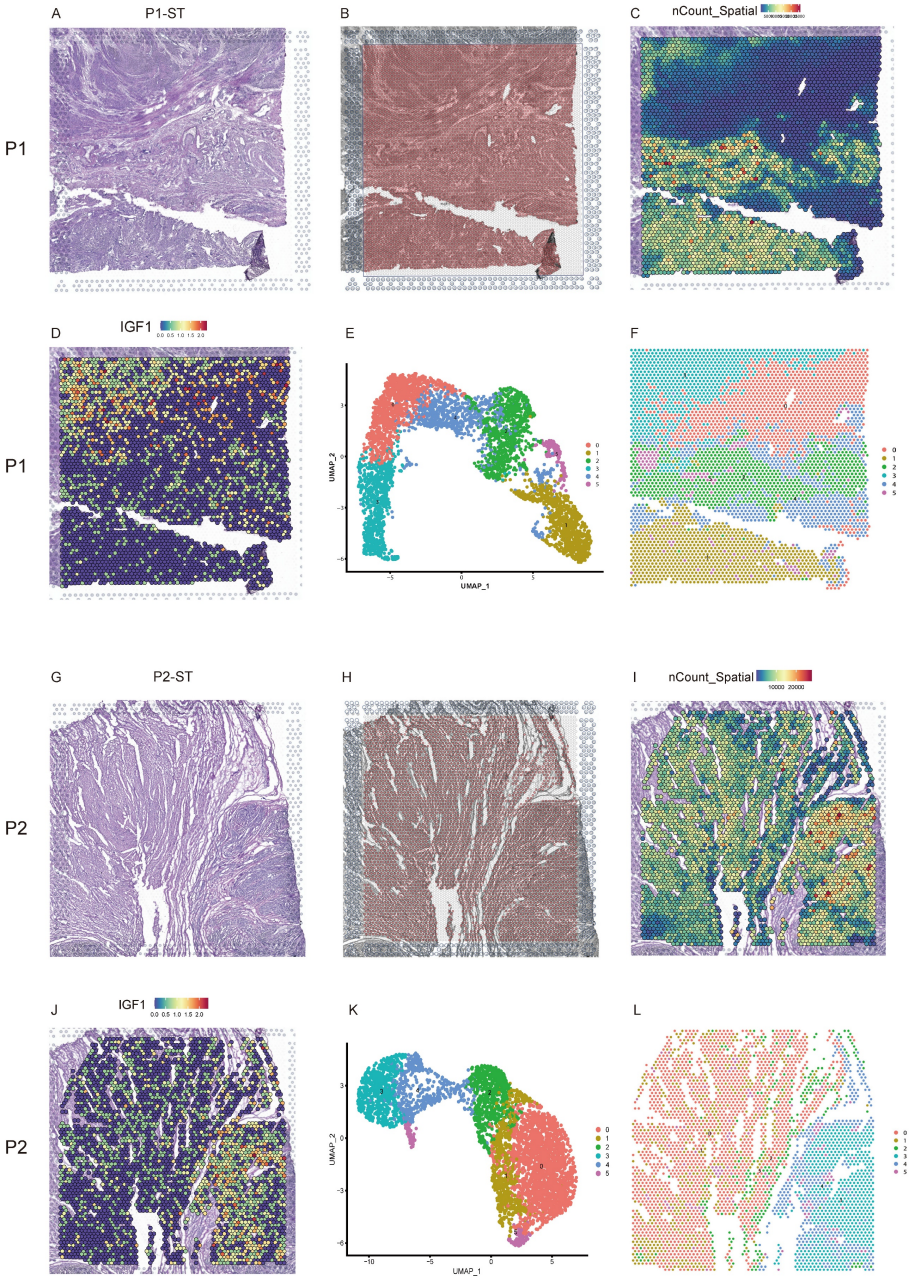
** The morphology and spatial transcriptomics of human uterine leiomyoma and its surrounding pseudocapsule. A.** Hematoxylin-eosin staining of P1-ST tissue section including areas of leiomyoma, its surrounding pseudocapsule and normal myometrium in the distance;** B.** The location of 5000 spots across P1-ST tissue section;** C.** The spatial expression patterns of all the genes in each spot of P1-ST tissue section;** D.** The spatial expression pattern of IGF1 in P1-ST tissue section;** E.** Hierarchical clustering of localized spots with UMAP;** F.** Unbiased clustering of ST spots in P1-ST; **G.** Hematoxylin-eosin staining of P2-ST tissue section including leiomyoma and its surrounding pseudocapsule;** H.** The location of 5000 spots across P2-ST tissue section;** I.** The spatial expression pattern of all the genes in each spot of P2-ST tissue section;** J.** The spatial expression pattern of IGF1 in P2-ST tissue section;** K.** Hierarchical clustering of localized spots with UMAP;** L.** Unbiased clustering of ST spots in P2-ST. P1, patient 1; P2, patient 2; P1-ST, sample 1 from patient 1 for spatial transcriptomics; P2-ST, sample 2 from patient 2 for spatial transcriptomics.

Uterine leiomyoma tissues were from the uteri after hysterectomy and subjected to freezing. The hematoxylin eosin (H&E)-stained tissue image of P1-ST exhibited the histological structure of leiomyoma, its surrounding pseudocapsule and normal myometrium in the distance from patient 1 (Figure 1A). Figure 1B exhibited the location of 5000 spots across P1-ST tissue section. The spatial expression pattern of all the genes in each spot of P1-ST was shown in Figure 1C and 4353 spots were detected on this sample section at a median depth of 2611 unique molecular identifiers (UMIs) with 1261 genes per spot. IGF1 expression was exhibited as representative in Figure 1D. ST spots were clustered by PCA and 6 clusters were acquired (Figure 1E). Next, the 6 clusters were mapped onto P1-ST section based on spot-unique spatial barcodes and the unbiased clustering of ST spots was shown in Figure 1F, which showed the similar shape with ST slide area. The H&E-stained tissue image of P2-ST showed the histological structure of leiomyoma and its surrounding pseudocapsule from patient 2 (Figure 1G). Figure 1H exhibited the location of 5000 spots across P2-ST tissue section. After data sequencing, we detected 4002 spots on this sample section at a median depth of 7877 UMIs with 2768 genes per spot (Figure 1I) and IGF1 expression was exhibited as representative in Figure 1J. ST spots were clustered by PCA and 6 clusters were also acquired (Figure 1K). The 6 clusters were mapped onto P2-ST section based on spot-unique spatial barcodes (Figure 1L). The above data successfully exhibited the overall morphology and spatial transcriptomics of human uterine leiomyoma, its surrounding pseudocapsule and normal myometrium.

### Single-cell transcriptional profiles of uterine leiomyoma and its surrounding pseudocapsule

**Figure 2:**
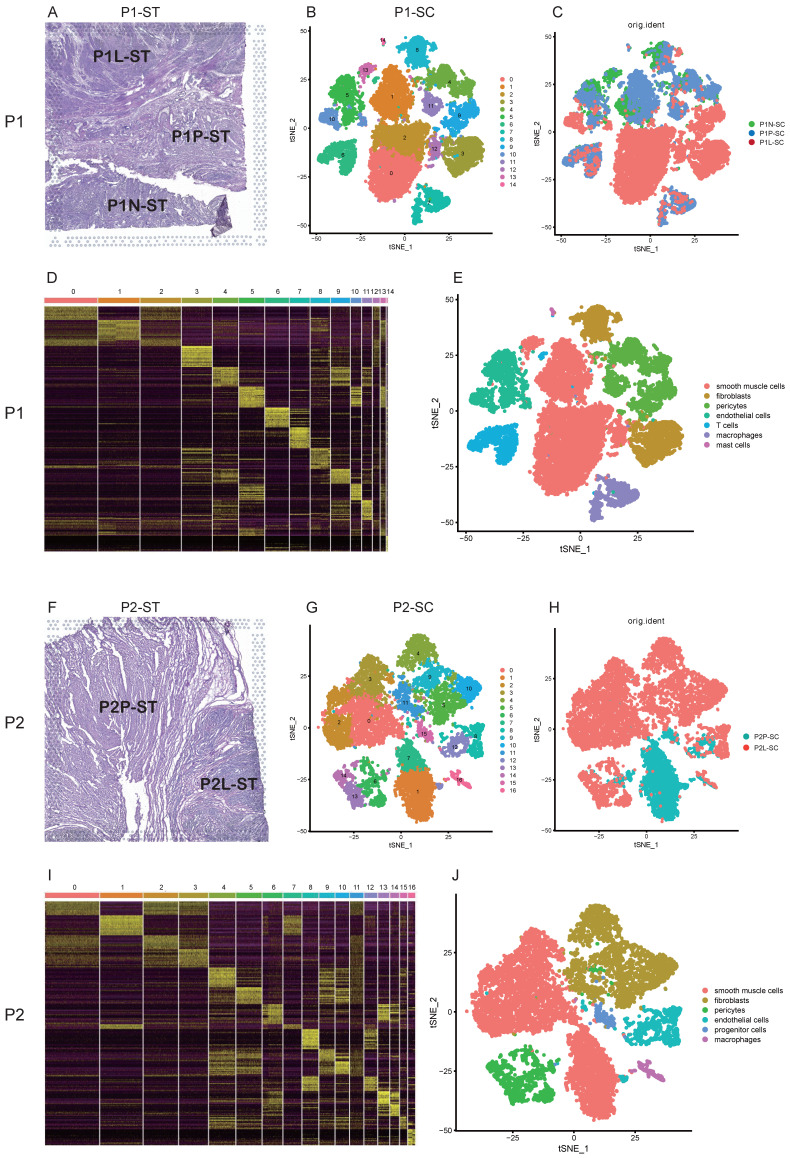
** Single-cell transcriptional profiles of uterine leiomyoma, surrounding pseudocapsule and myometrium from five samples of two patients. A.** Hematoxylin-eosin staining of P1-ST tissue section. It was divided into P1L-ST, P1P-ST and P1N-ST areas. The counterparts to P1L-ST, P1P-ST and P1N-ST were the adjacent samples of leiomyoma (P1L-SC), pseudocapsule (P1P-SC) and normal myometrium (P1N-SC) respectively and then were subjected to snRNA-seq; **B.** 2D visualization of 15 cell clusters of P1-SC (including P1L-SC, P1P-SC and P1N-SC) on the tSNE plot; **C.** The original identities of above 15 clusters; **D.** Heatmap plotting the top 20 differentially expressed marker genes for each cluster; **E.** 2D visualization of 7 annotated cell types on the tSNE plot of P1-SC; **F.** Hematoxylin-eosin staining of P2-ST tissue section. It was divided into P2L-ST and P2P-ST areas. The counterparts to P2L-ST and P2P-ST were adjacent samples of leiomyoma (P2L-SC) and pseudocapsule (P2P-SC) respectively and were then subjected to snRNA-seq; **G.** 2D visualization of 17 cell clusters of P2-SC (including P2L-SC and P2P-SC) on the tSNE plot; **H.** The original identities of above 17 clusters; **I.** Heatmap plotting the top 20 differentially expressed marker genes for each cluster;** J.** 2D visualization of 6 cell types on the tSNE plot of P2-SC. P1, patient 1; P2, patient 2; P1-ST, sample 1 from patient 1 for spatial transcriptomics; P2-ST, sample 2 from patient 2 for spatial transcriptomics.

For patient 1, these samples including leiomyoma, its surrounding pseudocapsule and normal myometrium in the distance were subjected to snRNA-seq (Figure 2A, C). A total of 15,289 cells were obtained after quality control and standardization. All cells were clustered by PCA and 15 clusters were generated, which were visualized by t-SNE (Figure 2B). Based on the expression pattern of the marker genes (Figure 2D), we divided these cell populations into 7 cell types, including smooth muscle cells (expressing ACTA2 and ACTG2), fibroblasts (expressing DCN), pericytes (expressing PDGFRB and NOTCH3), endothelial cells (expressing VWF and PECAM1), T cells (expressing ITK and CD2), macrophages (expressing MRC1), and mast cells (expressing KIT) (Figure S1). Clusters 0, 1, 2, 12 and 13 were annotated as smooth muscle cells, clusters 3 and 8 as fibroblasts, clusters 4, 9 and 11 as pericytes, clusters 5 and 10 as endothelial cells, cluster 6 as T cells, cluster 7 as macrophages, and cluster 14 as mast cells (Figure 2B, E). Here, we found the presence of cellular heterogeneity in smooth muscle cells and fibroblasts of uterine leiomyoma even in one patient since each cluster exhibited differential genes. For patient 2, two samples including leiomyoma and its surrounding pseudocapsule were subjected to snRNA-seq (Figure 2F, H). A total of 11,082 cells were obtained after quality control and standardization. 17 clusters were generated and visualized by t-SNE (Figure 2G). Based on the expression pattern of the marker genes (Figure 2I), 6 cell types were annotated, including smooth muscle cells (expressing ACTA2 and ACTG2), fibroblasts (expressing DCN), pericytes (expressing PDGFRB and NOTCH3), endothelial cells (expressing VWF and PECAM1), progenitor cells (expressing CD44 and ZFHX4) and macrophages (expressing MRC1) (Figure S2). Finally, clusters 0, 1, 2, 3 and 7 were annotated as smooth muscle cells, clusters 4, 5, 9, 10 and 11 as fibroblasts, clusters 6, 13 and 14 as pericytes, clusters 8 and 12 as endothelial cells, cluster 15 as progenitor cells, and cluster 16 as macrophages (Figure 2G, J). We also found the presence of cellular heterogeneity in smooth muscle cells and fibroblasts in uterine leiomyoma in this patient. The above data exhibited the overall transcriptional profiles of uterine leiomyoma, its surrounding pseudocapsule and normal myometrium at a single-cell resolution.

### Pseudotime analysis revealed the factors and signaling pathway that drived the occurrence and progression of uterine leiomyoma

**Figure 3:**
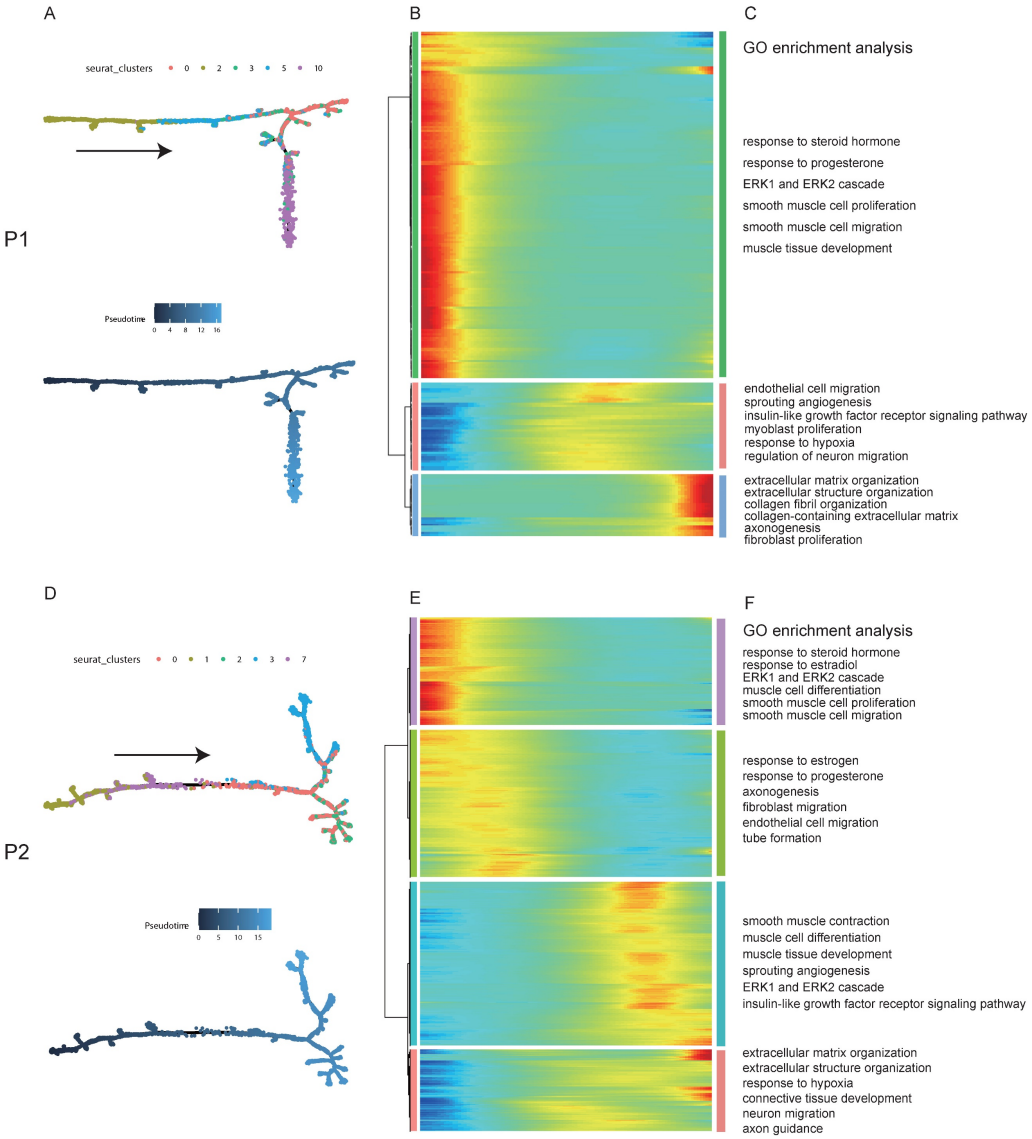
** The trajectory analysis of smooth muscle cells in leiomyoma and its surrounding pseudocapsule and Gene Ontology (GO) enrichment analysis during the evolutionary process. A.** Pseudotime single-cell trajectory was reconstructed by Monocle 2 for smooth muscle cells in leiomyoma (P1L-SC) and its surrounding pseudocapsule (P1P-SC). Smooth muscle cells in P1L-SC and P1P-SC included clusters 0, 2, 3, 5 and 10 shown in Figure S3A and C. Cells were labeled by seurat clusters (top) and pseudotime (bottom). **B.** Pseudotemporal heatmap exhibited gene expression dynamics for significant marker genes. Genes (rows) were clustered into three modules, and cells (columns) were ordered according to pseudotime; **C.** GO BP pathway of DEGs from 3 different clusters of left heatmap by enrichment analysis; **D.** Trajectory analysis of smooth muscle cells in leiomyoma (P2L-SC) and its surrounding pseudocapsule (P2P-SC) by Monocle 2. Cells were labeled by seurat clusters (top) and pseudotime (bottom). Smooth muscle cells in P2-SC included clusters 0, 1, 2, 3 and 7 shown in Figure 2G; **E.** Changes of DEGs of smooth muscle cells in pseudotime trajectory; **F.** GO BP pathway of DEGs in 4 different clusters of left heatmap by enrichment analysis. P1, patient 1; P2, patient 2; P1-SC, samples including leiomyoma (P1L-SC), pseudocapsule (P1P-SC) and normal myometrium (P1N-SC) from patient 1 for snRNA-seq; P2-SC, samples including leiomyoma (P2L-SC) and pseudocapsule (P2P-SC) from patient 2 for snRNA-seq; GO, Gene Ontology; BP, biological process.

To explore the dynamical evolution of leiomyoma cells, trajectory analysis of smooth muscle cells in leiomyoma and its surrounding pseudocapsule was performed. For patient 1, tSNE plot of uterine leiomyoma and surrounding pseudocapsule (without normal endometrium) was redrawn and defined, smooth muscle cells included clusters 0, 2, 3, 5, and 10 (Figure S3A and C). To obtain temporal resolution of these subclusters, we employed the pseudotemporal ordering algorithm Monocle 2 on clusters 0, 2, 3, 5, and 10. The cell trajectory started from cluster 2, followed with clusters 5 and 3, and ended with clusters 0 and 10 (Figure 3A).

Most of cells in cluster 2 were belonged to surrounding pseudocapsule (Figure S3B), indicating that the leiomyoma might derive from its surrounding pseudocapsule. The significantly changed genes among these five subclusters in pseudotime trajectory were examined and arranged into three gene modules according to their pseudotemporal expression patterns (Figure 3B). GO enrichment analysis of gene set in above modules showed that estrogen and progesterone were the possible driving factors that mediated the proliferation of smooth muscle cells and fibroblasts, formation of new blood vessels, extension of nerve fibers and organization of extracellular matrix (ECM) with possible involvement of ERK1/ERK2 pathway (Figure 3C). In patient 2, smooth muscle cells included clusters 0, 1, 2, 3 and 7, and most cells in clusters 1 and 7 were from surrounding pseudocapsule. Ordering of cells in pseudotime arranged most of muscle cells into a major trajectory and cells located in the start of pseudotime corresponded to cluster 1 (Figures 3D). We further checked pseudotime dynamics of significantly changed genes among these clusters and arranged them into four modules (Figures 3E). Gene ontology analysis of up-regulated genes further validated the results from patient 1 (Figures 3F). Collectively, pseudotime analysis indicated that estrogen and progesterone drived the occurrence and progression of uterine leiomyoma and ERK1/ERK2 signaling pathway might participate in the processes.

### Estrogen receptor alpha (ERα) and progesterone receptor (PGR) mediated the development of uterine leiomyoma possibly through ERK1/ERK2 signaling pathway

**Figure 4:**
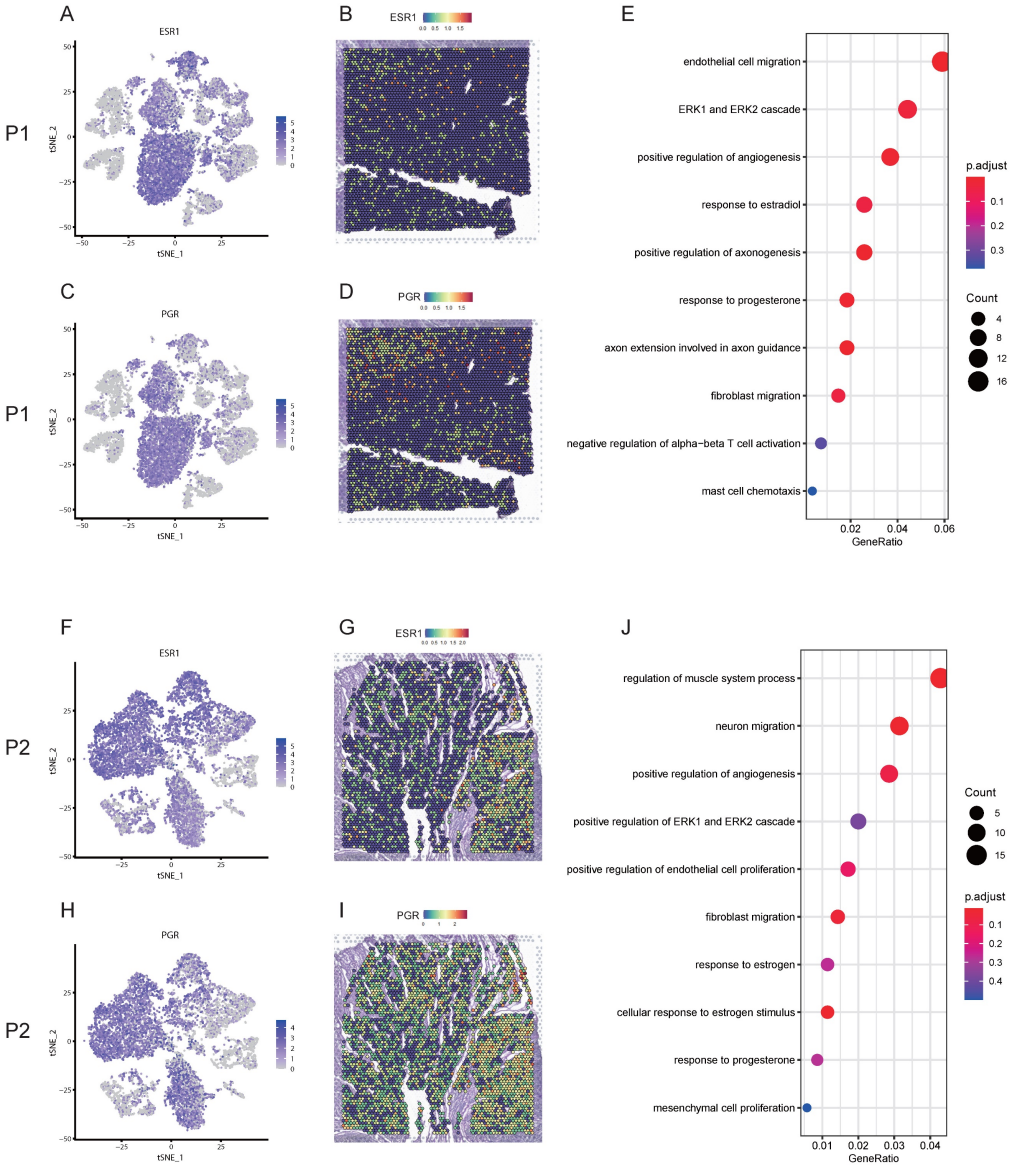
** The gene expression profiles and possible roles of ESR1 and PGR in the development of uterine leiomyoma. A.** The distribution of ESR1 on t-SNE plot of P1-SC; **B.** Spatial plots showed the spatial expression pattern of ESR1 in P1-ST; **C.** The distribution of PGR on t-SNE plot of P1-SC; **D.** Spatial plots showed the spatial expression pattern of PGR in P1-ST; **E.** GO enrichment analysis of DEGs in smooth muscle cells between pseudocapsule and leiomyoma of P1-SC; **F.** The distribution of ESR1 on t-SNE plot of P2-SC; **G.** Spatial plots showed the spatial expression pattern of ESR1 in P2-ST; **H.** The distribution of PGR on t-SNE plot of P2-SC; **I.** Spatial plots showed the spatial expression pattern of PGR in P2-ST; **J.** GO enrichment analysis of DEGs in smooth muscle cells between pseudocapsule and leiomyoma of P2-SC. P1, patient 1; P2, patient 2; P1-SC, samples including leiomyoma (P1L-SC), pseudocapsule (P1P-SC) and normal myometrium (P1N-SC) from patient 1 for snRNA-seq; P2-SC, samples including leiomyoma (P2L-SC) and pseudocapsule (P2P-SC) from patient 2 for snRNA-seq; GO, Gene Ontology; DEGs, differential expression genes.

Uterine leiomyoma is an estrogen and progesterone dependent disease. Here, we firstly checked the expression and location of estrogen and progesterone related genes at a single-cell and spatial resolution. For patient 1, estrogen receptor 1 (ESR1) was highly expressed in smooth muscle cells and fibroblasts based on snRNA-seq (Figure 4A). ST image showed that the expression of ESR1 was highly distributed in the tissue of leiomyoma (Figure 4B), indicating ERα might mediate the development of uterine leiomyoma. PGR was highly expressed in smooth muscle cells (Figure 4C) and ST image exhibited the main distribution of PGR in the tissue of leiomyoma (Figure 4D), indicating PGR played an important role in the development of uterine leiomyoma. GO enrichment analysis of DEGs in smooth muscle cells between pseudocapsule and leiomyoma of S1-SC (table S1) exhibiting the participation of estrogen, progesterone and ERK1/ERK2 pathway in the development of leiomyoma (Figure 4E). For patient 2, ESR1 was highly expressed in smooth muscle cells and fibroblasts (Figure 4F). ST image showed that the expression of ESR1 was mainly distributed in the tissue of leiomyoma (Figure 4G). PGR was highly expressed in smooth muscle cells and fibroblasts (Figure 4H) and ST image exhibited the high distribution of PGR in leiomyoma tissue (Figure 4I). GO enrichment analysis of DEGs in smooth muscle cells between pseudocapsule and leiomyoma of S2-SC (table S2) was conducted, showing that estrogen, progesterone and ERK1/ERK2 pathway might played roles (Figure 4J). Collectively, since ERK1/ERK2 lies in the downstream compared to the receptors of estrogen and progesterone, the above data indicated that estrogen and progesterone mediated the development of uterine leoimyoma possibly through ERK1/ERK2 signaling pathway, which further validated the results from the above pseudotime analysis.

### Estrogen receptor beta (ERβ) involved in the angiogenesis of uterine leiomyoma possibly via ERK1/ERK2 signaling pathway

**Figure 5:**
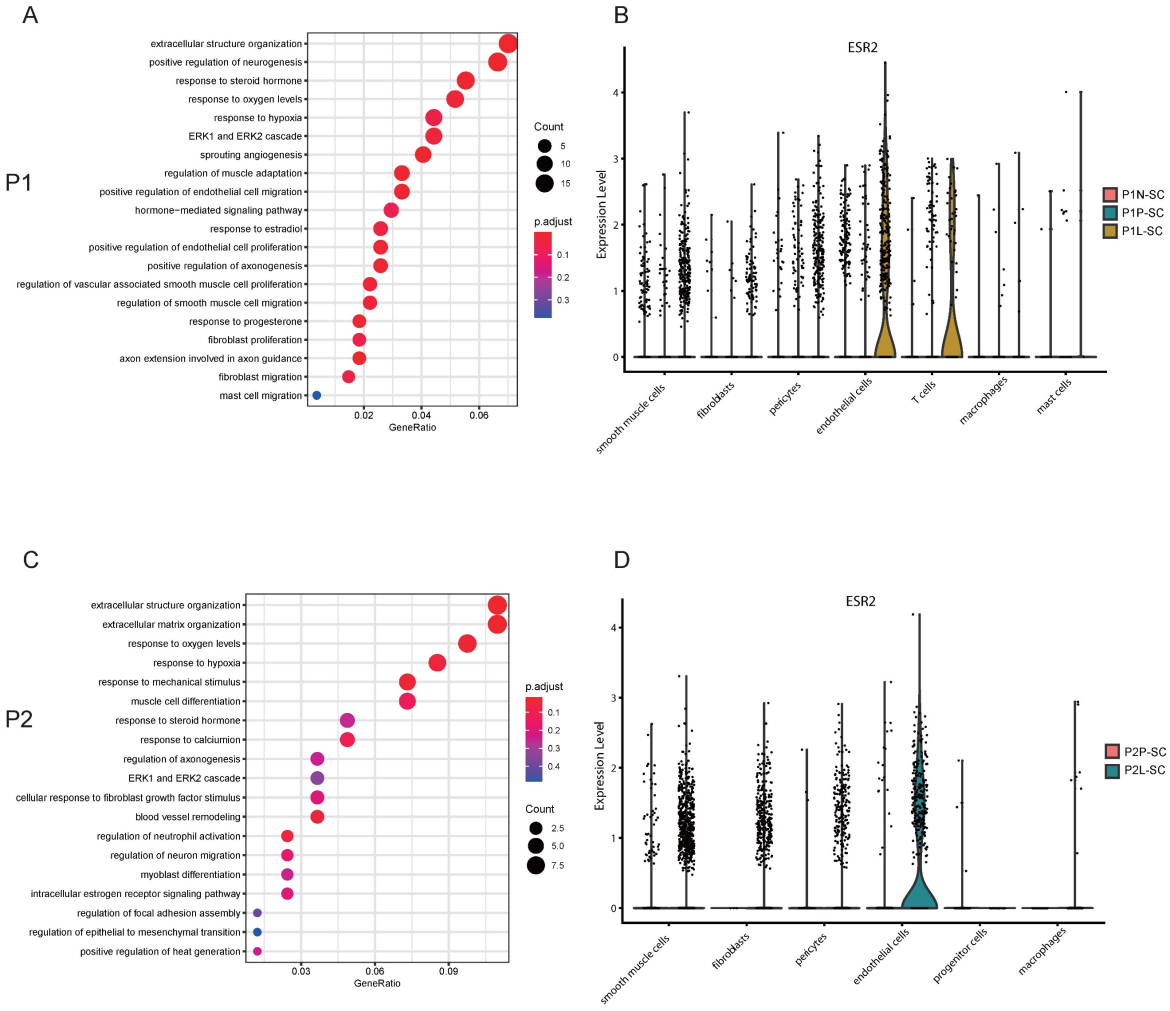
** ESR2 might involve in the angiogenesis of uterine leiomyoma. A.** GO enrichment analysis of DEGs in endothelial cells between pseudocapsule and leiomyoma of P1-SC; **B.** The expression of ESR2 in the located cells of leiomyoma, surrounding pseudocapsule and normal myometrium of P1-SC shown in violin plot; **C.** GO enrichment analysis of DEGs in endothelial cells between pseudocapsule and leiomyoma of P2-SC; **D.** The expression of ESR2 in the located cells of leiomyoma and surrounding pseudocapsule of P2-SC shown in violin plot. P1, patient 1; P2, patient 2; DEGs, differential expression genes. P1-SC, samples including leiomyoma (P1L-SC), pseudocapsule (P1P-SC) and normal myometrium (P1N-SC) from patient 1 for snRNA-seq; P2-SC, samples including leiomyoma (P2L-SC) and pseudocapsule (P2P-SC) from patient 2 for snRNA-seq. GO, Gene Ontology; DEGs, differential expression genes.

Angiogenesis is critical for the occurrence and development of leiomyoma. Blood vessels are composed of endothelial cells and pericytes 22. In P1-SC, endothelial cells included clusters 5 and 10, and DEGs were calculated. ESR1, estrogen receptor 2 (ESR2) and PGR were significantly up-regulated in endothelial cells of leiomyoma compared to pseudocapsule (table S3). GO enrichment analysis of DEGs in endothelial cells between pseudocapsule and leiomyoma showed that estrogen and ERK1/ERK2 signaling pathway might participate the formation of new blood vessels (Figure 5A). We found that the highest expression of ESR2 was in endothelial cells of leiomyoma (Figure 5B), indicating that ERβ might play an important role in the angiogenesis of uterine leiomyoma since uterine leiomyoma is an estrogen dependent disease. In addition, ESR2 was also expressed in T cells, hinting the function of T cells might be also regulated by estrogen, but the specific mechanisms still need further investigation. In P2-SC, endothelial cells included clusters 8 and 12, and ESR2 was significantly up-regulated in endothelial cells of leiomyoma compared to pseudocapsule (table S4). Similar results were obtained from the GO enrichment analysis of DEGs in endothelial cells between pseudocapsule and leiomyoma (Figure 5C) and ESR2 was mainly expressed in endothelial cells of leiomyoma in P2-SC (Figure 5D). The data showed that estrogen/ERβ involved in the angiogenesis of uterine leiomyoma possibly via ERK1/ERK2 signaling pathway. Collectively, our results revealed potentially feasible strategies for hormonotherapy and non-hormonal targeted therapy in uterine leiomyoma.

### Cell-cell communications were common in the interior of leiomyoma and its surrounding pseudocapsule and IGF1-IGF1R might serve as a therapeutic target for non-hormonal treatment

**Figure 6:**
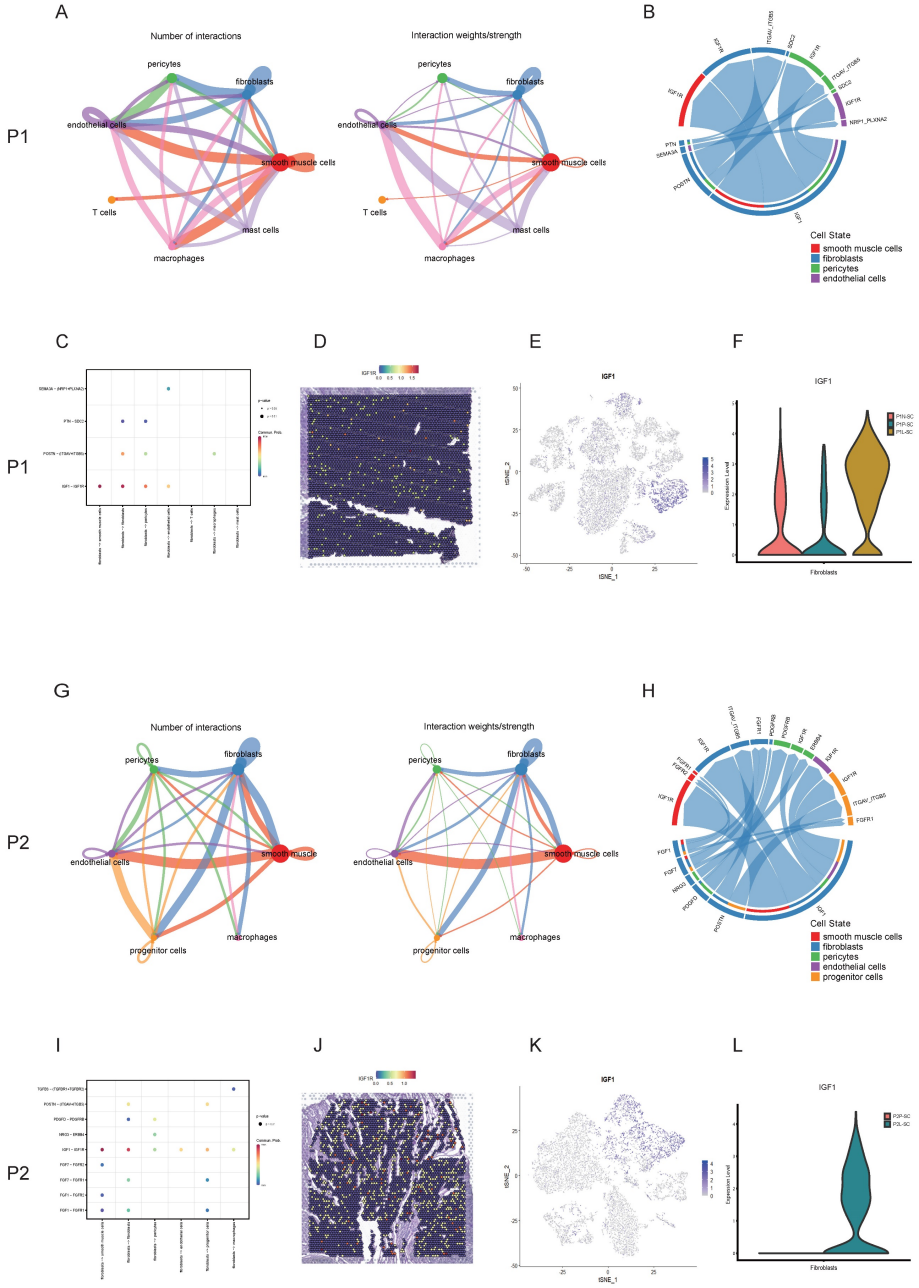
** Cell-cell interactions between leiomyoma and its surrounding pseudocapsule and in the interior of leiomyoma. A.** The quantity and strength of cell-cell interactions among any two cell types in P1-SC shown in circle plots; **B.** The ligand-receptor pairs between two cells were visualized by chord diagram; **C.** The ligand-receptor pairs between fibroblasts and other cells were further detailed by bubble plot; **D.** The spatial expression pattern of IGF1R in P1-ST on spatial plots; **E.** The IGF1 distribution of P1-SC on t-SNE plot; **F.** The IGF1 expression levels in fibroblasts of P1N-SC, P1P-SC and P1L-SC on violin plot; **G.** The quantity and strength of cell-cell interactions among any two cell types in P2-SC shown in circle plots; **H.** The ligand-receptor pairs between two cells were visualized by chord diagram; **I.** The ligand-receptor pairs between fibroblasts and other cells were further detailed by bubble plot; **J.** The spatial expression pattern of IGF1R in P2-ST on spatial plots; **K.** The IGF1 distribution in P2-SC on t-SNE plot; L. The IGF1 expression levels in fibroblasts of P2P-SC and P2L-SC on violin plot. P1, patient 1; P2, patient 2; P1-SC, samples including leiomyoma (P1L-SC), pseudocapsule (P1P-SC) and normal myometrium (P1N-SC) from patient 1 for snRNA-seq; P2-SC, samples including leiomyoma (P2L-SC) and pseudocapsule (P2P-SC) from patient 2 for snRNA-seq.

Uterine leiomyoma is not an isolated tumor, cell-cell communications happen in the interior of leiomyoma and with its surrounding pseudocapsule at every moment. To find out the contents and methods of communications, CellChat analysis was applied. For P1-SC, circle plots showed the quantity and strength of cell-cell interactions among any two cell types (Figure 6A), which exhibited that fibroblasts communicated widely with other cells. Next, chord diagram displayed that IGF1-IGF1R was the most significant ligand-receptor pair (Figure 6B). Bubble plot further showed fibroblasts interacted with other three cells (smooth muscle cells, endothelial cells and pericytes) mainly through IGF1-IGF1R (Figure 6C), indicating that fibroblast-secreted IGF1 might play an important role in the progression of uterine leiomyoma of patient 1. The spatial expression pattern of IGF1R on spatial plots in P1-ST was further checked (Figure 6D). IGF1 was mainly expressed in fibroblasts (Figure 6E) while the IGF1 expression level in leiomyoma associated fibroblasts was significantly higher than these in surrounding pseudocapsule and normal myometrium (Figure 6F). For P2-SC, circle plots also showed the quantity and strength of cell-cell communications (Figure 6G). Among them, the interaction between fibroblasts and other cells was a significant one. Chord diagram showed IGF1-IGF1R was the most significant ligand-receptor pair (Figure 6H). Bubble plot further showed fibroblasts interacted with other cells mainly via IGF1-IGF1R (Figure 6I), indicating that fibroblast-secreted IGF1 might play an important role in the progression of uterine leiomyoma of patient 2. The spatial expression pattern of IGF1R on spatial plots in P2-ST was also checked (Figure 6J). IGF1 was mainly expressed in fibroblasts (Figure 6K) while the IGF1 expression level in leiomyoma-associated fibroblasts was significantly higher than that in surrounding pseudocapsule (Figure 6L). The above data showed that cell-cell communications were common and fibroblast-derived IGF1 might play an important role in the progression of uterine leiomyoma, which could serve as a therapeutic target for non-hormonal treatment.

### Integrating spatial transcriptomics with single-cell transcriptomics revealed effective strategies for reducing the bleeding risk and surgery skills during myomectomy

**Figure 7:**
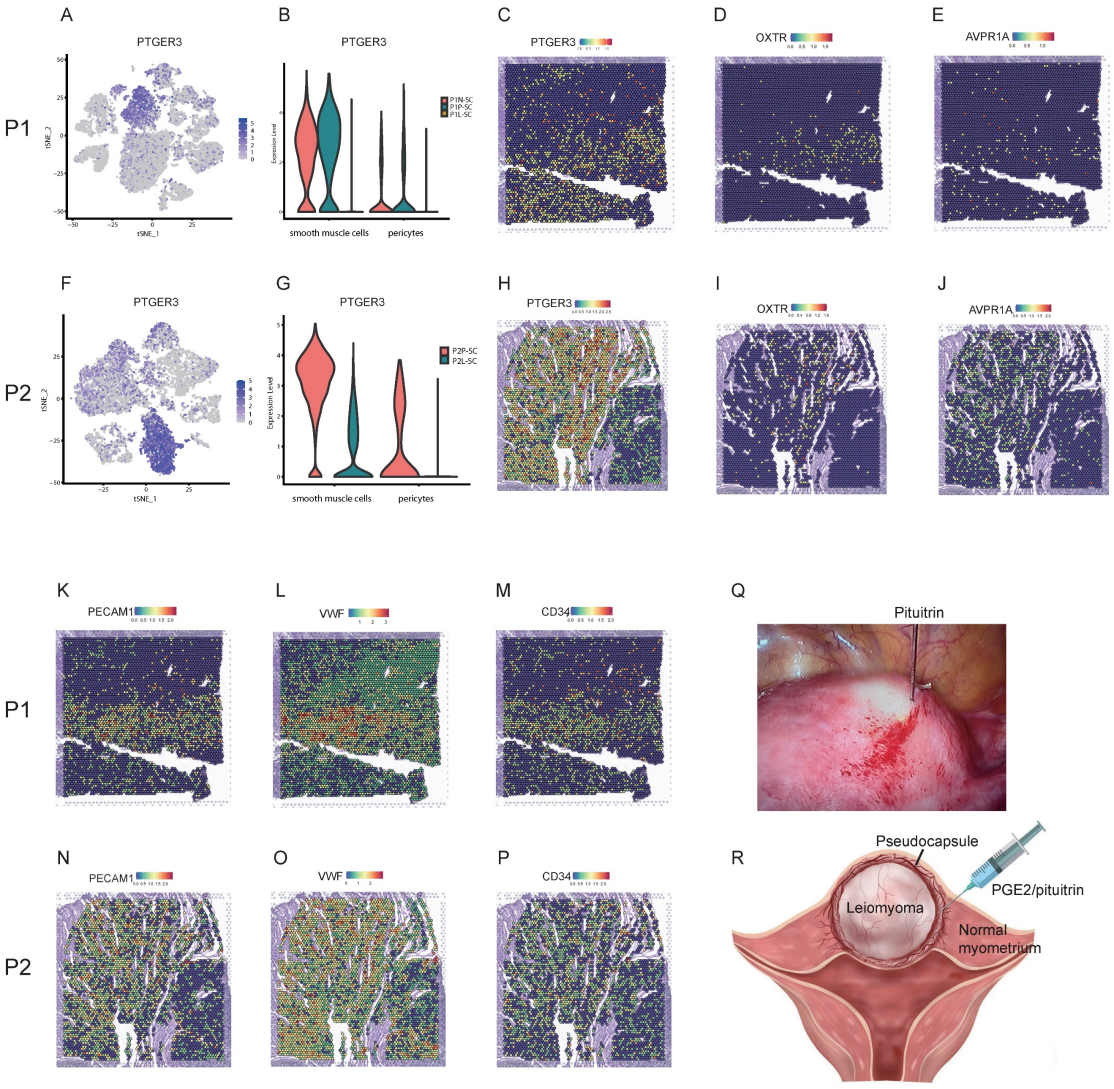
** Strategies for effective bleeding control during myomectomy. A.** The distribution of PTGER3 on t-SNE plot of P1-SC; **B.** The expression levels PTGER3 in smooth muscle cells and pericytes on violin plot of P1-SC; **C.** Spatial plots showed the spatial expression pattern of PTGER3 in P1-ST; The spatial expression patterns of OXTR (**D**), AVPR1A (**E**), PECAM1 (**K**), VWF (**L**) and CD34 (**M**) on spatial plots in P1-ST; **F.** The distribution of PTGER3 on t-SNE plot of P2-SC; **G.** The expression levels of PTGER3 in smooth muscle cells and pericytes on violin plot of P2-SC; **H.** Spatial plots show the spatial expression pattern of PTGER3 in P2-ST; The spatial expression patterns of OXTR (**I**), AVPR1A (**J**), PECAM1 (**N**), VWF (**O**) and CD34 (**P**) on spatial plots in P2-ST; **Q.** 6 units of pituitrin was injected into the junction between pseudocapsule and leiomyoma during laparoscopic myomectomy; **R.** Schematic diagram of PGE2/pituitrin injection into the junction between pseudocapsule and leiomyoma during myomectomy. P1, patient 1; P2, patient 2; P1-SC, samples including leiomyoma (P1L-SC), pseudocapsule (P1P-SC) and normal myometrium (P1N-SC) from patient 1 for snRNA-seq; P2-SC, samples including leiomyoma (P2L-SC) and pseudocapsule (P2P-SC) from patient 2 for snRNA-seq. PTGER3, prostaglandin E receptor 3; OXTR, oxytocin receptor; AVPR1A, arginine vasopressin receptor 1A; PECAM1, platelet and endothelial cell adhesion molecule 1; VWF, von Willebrand factor; PGE2, prostaglandin E2.

Myomectomy is the most common therapy method for patients with symptomatic leiomyoma who wish to preserve their uteri. However, bleeding control during surgery is still a challenge especially for the laparoscopic myomectomy. The single-cell transcriptomics showed that the expression level of prostaglandin E receptor 3 (PTGER3), one of top DEGs, was relatively high in smooth muscle cells and pericytes of surrounding pseudocapsule while was significantly down-regulated in the leiomyoma tissue in P1-SC and P2-SC (Figure 7A, B, F, G), which was further validated by spatially resolved analysis in both P1-ST and P2-ST (Figure 7C, H). Since the contractions of uterine smooth muscle cells and pericytes can reduce the bleeding from blood vessels of uterus, we inferred that prostaglandin E2 (PGE2) could be used as a vasoconstrictive agent to reduce the bleeding risk during myomectomy. The data also indicated that PGE2 should be injected into the junction of pseudocapsule and leiomyoma instead of leiomyoma tissue based on the following reasons. Firstly, the pseudocapsules were filled with blood vessels (Figure 7K, L, M, N, O and P). Secondly, PTGER3 was highly expressed on the smooth muscle cells in pseudocapsule compared to leiomyoma. Thirdly, water-pillow could be formed and then contribute to the separation of leiomyoma from its surrounding tissue. Fourthly, the junction of pseudocapsule and leiomyoma was quite loose, which was conducive to the infiltration of uterotonic agents around the leiomyoma and into the pseudocapsule. Fifthly, our previous study proved that nerve fibers were scattered among the pseudocapsule and had the similar distribution pattern with its adjacent normal myometrium 23. Sixthly, not too many significant differences were found between the pseudocapsule and normal myometrium regarding to the gene expressions of smooth muscle cells (table S5). Hence, the surrounding pseudocapsule should not be eliminated. Furthermore, since pituitrin can also induce uterine contraction and it contains oxytocin and vasopressin, we checked the expressions and locations of oxytocin receptor (OXTR) and arginine vasopressin receptor 1A (AVPR1A). ST images showed that OXTR and AVPR1A were highly expressed in pseudocapsule area instead of leiomyoma area (Figure 7D, E, I, and J). Collectively, the results demonstrated that PGE2 or pituitrin could be applied during the myomectomy, the injection site should be located at the junction of pseudocapsule and leiomyoma and the pseudocapsule should be reserved (Figure 7Q, and R). Therefore, our data revealed the effective strategies for bleeding control and surgery skills during myomectomy.

## Discussion

Bulk RNA-sequencing has been widely used to sequence RNA transcripts from a mixture of whole tissues and thereby it profiles average gene expression of all the included cells 10. ScRNA-seq restores the lost information about cellular heterogeneity in bulk RNA-sequencing and profiles single-cell transcriptomes while the spatial information is still lacking. ST could solve the above drawbacks, but it also has limitations in the resolution and depth. Therefore, the integration of scRNA-seq and ST can maximize mutual benefits and has advanced the depth and width of basic research in many diseases 10, 24. However, ST as an advanced technology has not being applied in the basic research of uterine leiomyoma, not even to mention the integration of scRNA-seq and ST. Here, we first comprehensively provided a large-scale and high-dimensional atlas of ST and single-cell transcriptomics of uterine leiomyoma, its surrounding pseudocapsule and normal myometrium.

Successful scRNA-Seq of clinical specimens still poses challenges such as changes in gene expression and loss of sensitive cells due to immediate enzymatic digestion of fresh samples. snRNA-Seq can settle above challenges while also have its own drawbacks including the loss of genes in the cytoplasm. As for frozen and hard-to-dissociate samples, snRNA-Seq is thought to be a better method 25. During the process of ST, tissue samples are frozen and the slides with targeted area need to be carefully selected for the following sequencing. To better obtain the matched sample, the adjacent area was the optimal one for single cell sequencing. Since snRNA-Seq is usually performed after successful ST sequencing and thereby can profile the accurate counterparts with ST tissues. In this study, snRNA-Seq was applied and charted the tumor ecosystems consisting of leiomyoma cells, myometrial cells, endothelial cells, pericytes, fibroblasts, macrophages, T cells, progenitor cells and mast cells, which was basically consistent with previous study 8, indicating snRNA-Seq is a qualified tool for samples from the uterus. Our data also further proved the cellular heterogeneity in uterine leiomyoma including leiomyoma cells, endothelial cells, pericytes and fibroblasts.

Estrogen has been traditionally considered as the major promoter of uterine leiomyoma while new theory believes that progesterone is also critical for the multistep cascade of leiomyoma growth 26. Later, epidemiological investigations, clinical trials and experimental data have highlighted the role of estrogen and progesterone in the pathogenesis and progression of uterine leiomyoma 27. Although the exact mechanism of how ovarian steroid hormones drive the tumorigenesis of leiomyoma is still unclear, estrogen receptors and progesterone receptor are indispensable during this process. Here, both snRNA-seq and ST results showed that ESR1 and PGR were expressed higher in leiomyoma than these in pseudocapsule and myometrium. Pseudotime analysis revealed that estrogen and progesterone might drive the occurrence and development of uterine leiomyoma and GO enrichment analysis validated that ERK1/ERK2 signaling pathway might be a critical participator during the process. Angiogenesis is an important step for the growth of uterine leiomyoma and blood vessels are composed of endothelial cells and pericytes 22. Our snRNA-seq data showed that ESR2 was mainly expressed in leiomyoma endothelial cells and Valladares et al. aslo reported that ERβ was highly located on the leiomyoma endothelial cells. Since ERβ could promote angiogenesis 28, 29, ERβ might mediate the angiogenesis of uterine leiomyoma. GO enrichment analysis of DEGs in endothelial cells between pseudocapsule and leiomyoma showed that estrogen and progesterone might mediate the migration and proliferation of endothelial cells and ERK1/ERK2 signaling pathway also participated in the angiogenesis. Since ERK1/ERK2 signaling pathway is critical in the tumor angiogenesis 30, 31, we inferred that ERβ might involve in the angiogenesis via ERK1/ERK2 signaling pathway, which might serve as a non-hormonal therapy target. Therefore, we further validated the effectiveness of hormonal therapy in the treatment of uterine leiomyoma and ERK1/ERK2 might serve as a therapeutic target from the perspectives of snRNA-seq and ST.

Cell-cell communications are common not only in cancers but also in benign tumors including uterine leiomyoma. Cellchat analysis found that leiomyoma cells were closely connected with other cells in the interior of leiomyoma and its surrounding pseudocapsule. Among them, IGF1-IGF1R was the main ligand-receptor pairs in the interactions between fibroblasts and other cells. Under the effects of estrogen and progesterone, leiomyoma cells could promote the migration and proliferation of fibroblasts, while fibroblasts contributed to the development of leiomyoma in return via the secretion of IGF1. Furthermore, ST images showed fibroblasts were intertwined with leiomyoma cells. In addition, leiomyoma cells and fibroblasts are also responsible for the accumulation of ECM and ECM is an important component part of leiomyoma 32. ECM could serve as a critical therapeutic target to control the abnormal growth of leiomyoma 33. We found that leiomyoma-associated fibroblasts could secrete ECM including collagen, fibronectin and ADAM19 (Figure S4, 5). Therefore, leiomyoma-associated fibroblasts might play an important role in the progression of uterine leiomyoma via IGF1-IGF1R. Furthermore, progenitor cells were defined and we thought they were myometrial progenitor cells, which was stated by Maruyama et al. in a previous report 34. They proposed that myometrial progenitor cells might play a role in pathogenesis of leiomyoma but it still needed further validation. Collectively, our results explained why hormonotherapy was effective and further provided therapeutic targets including ERK1/ERK2 signaling pathway and IGF1-IGF1R for non-hormonal therapy.

Besides drug therapy, myomectomy is another standard treatment of symptomatic leiomyoma for patients who wish to preserve uterus 35. The important thing to note here is the heavy bleeding especially during laparoscopic myomectomy and many methods have been used for the bleeding control such as interventions on uterine arteries, utero-tonics, myoma dissection techniques, pharmacologic manipulation of the coagulation cascade with antifibrinolytic agents. Among them, moderate-quality evidences support that vasopressin injection or intravaginal misoprostol may reduce bleeding during surgery, and low-quality evidence for other methods 36. Here, the analysis of snRNA-seq and ST showed that AVPR1A and OXTR were highly expressed in the pseudocapsule while down-regulated in the leiomyoma and that the pseudocapsule was filled with blood vessels, which explained why pituitrin take effects and indicated the optimal injection site should be located at the junction between pseudocapsule and leiomyoma. In our clinical practice, the injection of pituitrin into the junction of pseudocapsule and leiomyoma effectively reduced haemorrhage during myomectomy. Besides AVPR1A and OXTR, we further found PTGER3, one of top DEGs, was highly expressed on the smooth muscle cells in pseudocapsule compared to leiomyoma, indicated that PGE2 might be another candidate. Here, we first presented that PGE2 could be applied for bleeding control during myomectomy and the injection site should be located at the junction between pseudocapsule and leiomyoma, but it still needed the validation by randomized controlled trials. In addition, the similar distribution pattern of nerve fibers between pseudocapsule and its adjacent normal myometrium was reported by our previous study 23, indicating that the surrounding pseudocapsule should not be removed. Therefore, the results revealed the effective strategies for bleeding control and surgery skills during myomectomy.

Overall, the integration of spatial transcriptomics and single-nucleus RNA sequencing reveals the potential therapeutic strategies for uterine leiomyoma, which sets a direction for future research. Hormonotherapy such as the injection of gonadotrophin-releasing hormone agonist (GnRHa) monthly has been used for uterine leiomyomas, but the efficacy, side effects and long-term application require further high-quality clinic trials. Likewise, therapeutic targets including ERK1/ERK2 pathway and IGF1-IGF1R still need the validation from solid experimental evidences. As for the efficacy of prostaglandin E2 for bleeding control, we are planning to conduct a randomized controlled trial to investigate if it could be a better option for the gynecologists during myomectomy.

## Conclusions

A comprehensive transcriptional atlas of cellular architectures for uterine leiomyoma and pseudocapsule were exhibited by integration of snRNA-seq and ST at the first time. ERα and PGR mediated the occurrence and development of uterine leiomyoma and ERβ involved in the angiogenesis, which explained the effectiveness of hormonotherapy. Cell-cell communications were common in the interior of leiomyoma and its surrounding pseudocapsule. Therapeutic targets including ERK1/ERK2 pathway and IGF1-IGF1R might be applied for non-hormonal therapy. Injection of prostaglandin E2 was initially presented for bleeding control, the injection site should be located at the junction between pseudocapsule and leiomyoma, and the surrounding pseudocapsule should not be eliminated during myomectomy. Collectively, the results revealed the potentially feasible strategies for hormonotherapy, non-hormonal targeted therapy, and bleeding control during myomectomy.

## Supplementary Material

Supplementary figures and table legends.

Supplementary table 1.

Supplementary table 2.

Supplementary table 3.

Supplementary table 4.

Supplementary table 5.

## Abbreviations

ST: Spatial transcriptomics
SnRNA-seq: Single-nucleus RNA sequencing
ERα: Estrogen receptor alpha
PGR: Progesterone receptor
ERβ: Estrogen receptor beta
ScRNA-seq: Single-cell RNA sequencing
H&E: Hematoxylin-eosin
BSA: Bovine serum albumin
HBSS: Hanks' balanced salt solution
PCA: Principal component analysis
t-SNE: t-distributed stochastic neighbor embedding
GEMs: Gel bead-in-emulsion
DEGs: differentially expressed genes
GO: Gene Ontology
UMIs: unique molecular identifiers
ECM: extracellular matrix
ESR1: estrogen receptor 1
ESR2: estrogen receptor 2
PTGER3: prostaglandin E receptor 3
PGE2: prostaglandin E2
OXTR: oxytocin receptor
AVPR1A: arginine vasopressin receptor 1A
