# Supplementary material for: Integrating Spatial Transcriptomics and Single-nucleus RNA Sequencing Reveals the Potential Therapeutic Strategies for Uterine Leiomyoma
Source: Int J Biol Sci. 2023 May 8;19(8):2515–30. doi: 10.7150/ijbs.83510 (PMC10197899; doi:10.7150/ijbs.83510)

Supplementary information

Supplementary figure legends

Figure S1. The expression patterns of marker genes on t-SNE plot of P1-SC. The marker genes including ACTA2 and ACTG2 for smooth muscle cells, DCN for fibroblasts, PDGFRB and NOTCH3 for pericytes, VWF and PECAM1 for endothelial cells, ITK and CD2 for T cells, MRC1 for macrophages, and KIT for mast cells. P1-SC, samples including leiomyoma (P1L-SC), pseudocapsule (P1P-SC) and normal myometrium (P1N-SC) from patient 1 for snRNA-seq.

Figure S2. The expression patterns of marker genes on t-SNE plot of P2-SC. The marker genes including ACTA2 and ACTG2 for smooth muscle cells, DCN for fibroblasts, PDGFRB and NOTCH3 for pericytes, VWF and PECAM1 for endothelial cells, CD44 and ZFHX4 for progenitor cells, MRC1 for macrophages. P2-SC, samples including leiomyoma (P2L-SC) and pseudocapsule (P2P-SC) from patient 2 for snRNA-seq.

Figure S3. Single-cell transcriptional profiles of uterine leiomyoma and surrounding pseudocapsule from patient 1. A. 2D visualization of 15 cell clusters P1L-SC and P1P-SC on the tSNE plot; **B.** The original identities of above 15 clusters; **C.** 2D visualization of 7 annotated cell types on the tSNE plot of P1L-SC and P1P-SC.

Figure S4. The expression patterns of collagen genes on t-SNE plots of P1-SC. The distributions of COL1A1, COL1A2, COL3A1, COL4A1, COL5A2, COL6A2, COL6A3, COL7A1, COL12A1, COL16A1, FN1 and ADAM19 on t-SNE plot of P1-SC.

Figure S5. The expression patterns of collagen genes on t-SNE plots of P2-SC.

The distributions of COL1A1, COL1A2, COL3A1, COL4A1, COL5A2, COL6A2, COL6A3, COL7A1, COL12A1, COL16A1, FN1 and ADAM19 on t-SNE plot of P2-SC.

Supplementary table titles

Table S1. The differentially expressed genes in smooth muscle cells between pseudocapsule and leiomyoma of P1-SC.

Table S2. The differentially expressed genes in smooth muscle cells between pseudocapsule and leiomyoma of P2-SC.

Table S3. The differentially expressed genes in endothelial cells between pseudocapsule and leiomyoma of P1-SC.

Table S4. The differentially expressed genes in endothelial cells between pseudocapsule and leiomyoma of P2-SC.

Table S5. The differentially expressed genes in endothelial cells between normal myometrium and pseudocapsule of P1-SC.

Figure S1. The expression patterns of marker genes on t-SNE plot of P1-SC.

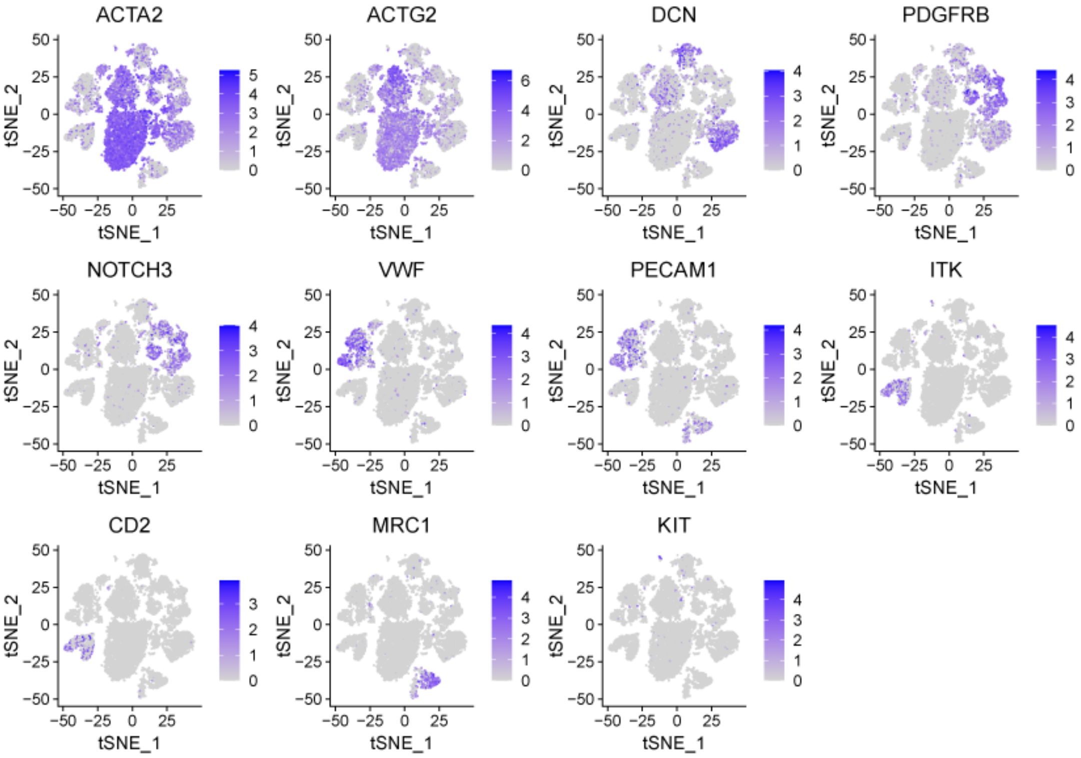

Figure S2. The expression patterns of marker genes on t-SNE plot of P2-SC.

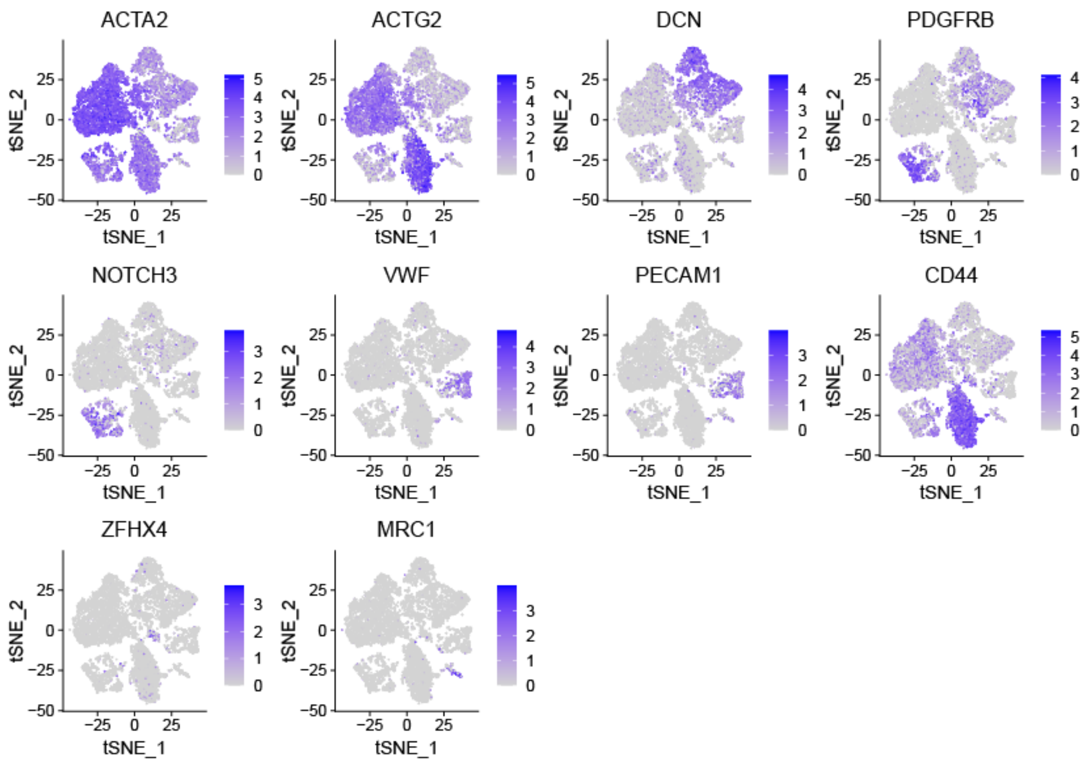

Figure S3. Single-cell transcriptional profiles of uterine leiomyoma and surrounding pseudocapsule from patient 1.

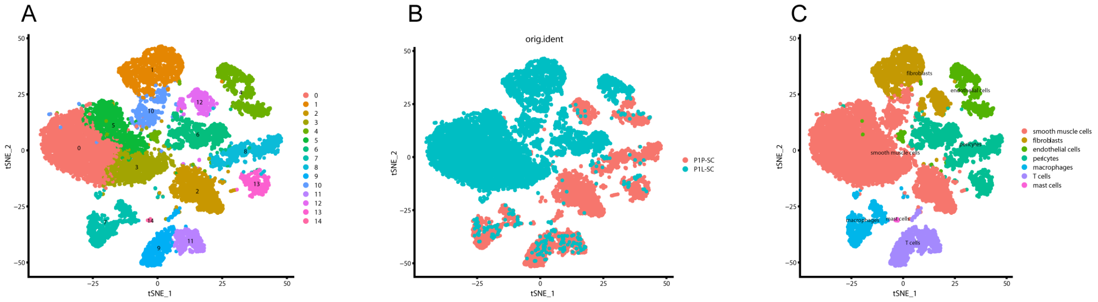

Figure S4. The expression patterns of collagen genes on t-SNE plots of P1-SC.

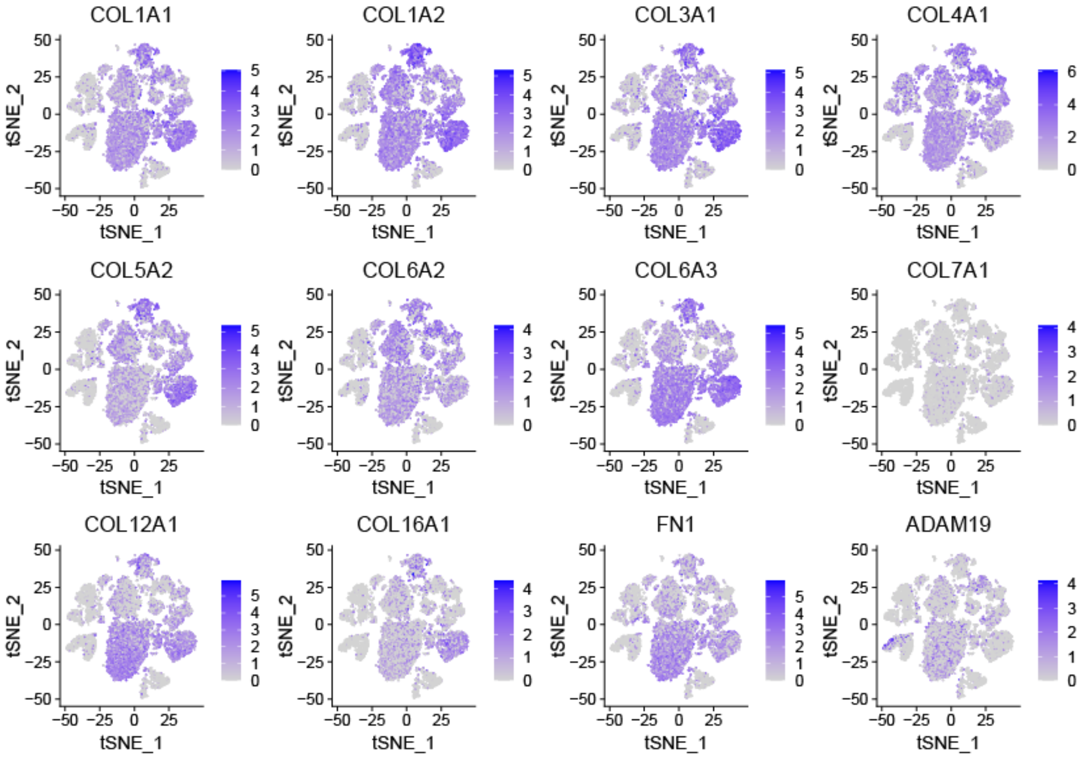

Figure S5. The expression patterns of collagen genes on t-SNE plots of P2-SC.

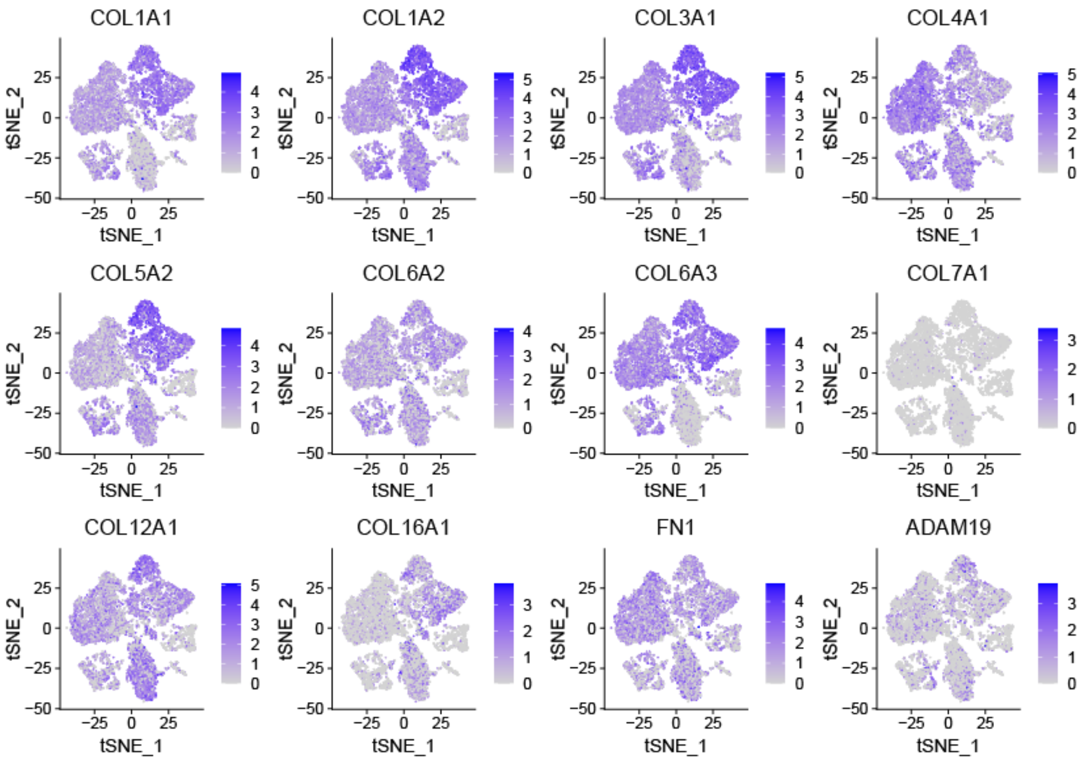

Supplement: Supplementary file 1 — Supplementary figures and table legends. [file ijbsv19p2515s1.pdf]
